# Vest-over-Pant Method for Closure of Residual Cavity of Liver Hydatid Cyst

**DOI:** 10.1155/2012/628176

**Published:** 2012-04-08

**Authors:** Saba Behdad, Akbar Behdad, Samin Behdad, Mehrdad Hosseinpour

**Affiliations:** ^1^Division of Animal Sciences, Isfahan University of Technology, Isfahan 81738, Iran; ^2^Department of General Surgery, Al Zahra Hosiptal, Medical University of Isfahan, Isfahan 88732, Iran; ^3^Trauma Research Center, Kashan University of Medical Sciences, Kashan 86654, Iran

## Abstract

*Objective*. Although several therapeutic strategies have proven to be effective for hydatid cyst of liver, but surgery is still the most common therapy despite its morbidity and mortality. Furthermore, a variety of technique has been recommended for managing the residual cavity after cystectomy. We report here a new technical method for the reconstruction of hydatid cyst residual cavity with using overlapping flaps of liver edges (Vest over Pant). *Methods*. In this technique after removing the cyst, the edges of one side of cyst cavity were sutured to the base of the cavity using three to four mattress sutures), and edges of other side of liver was overlapped on the dorsal part of previous layer using four to five mattress sutures. Therefore residual cavity dead space was obliterated with two surfaces of cavity. *Results*. Fifty males were treated by our method. The average cyst volume was 423 ± 110 mL. There was no intraabdominal sepsis, bile leakage, or hepatic necrosis. In follow-up ultrasound study, residual cavities were disappeared one month after operation. *Conclusion*. Overlapping flaps of liver edges (Vest over Pant) provides easy, safe closure of cyst with preservation of the liver anatomy.

## 1. Introduction

 The appropriate treatment for hydatid cyst of the liver [1] is determined by several factors such as the patient, the cyst, the therapeutic resources, and the physician. Although several therapeutic strategies have proven to be effective for hydatid cyst of liver [2, 3], surgery is still the most common therapy despite its morbidity and mortality [4]. Furthermore, a variety of techniques have been recommended for managing the residual cavity after cystectomy [5–11], but none of these methods is universally applicable [12]. We report a modified technique for introflexion of hydatid cyst residual cavity with using overlapping flaps of liver edges (vest over pant).

## 2. Method

This clinical prospective study was approved by the ethical committee of our university. Participants were all patients consecutively admitted to the general surgery unit of the study hospital for elective surgery of liver hydatid cyst.

### 2.1. Operative Technique

**Figure 1:**
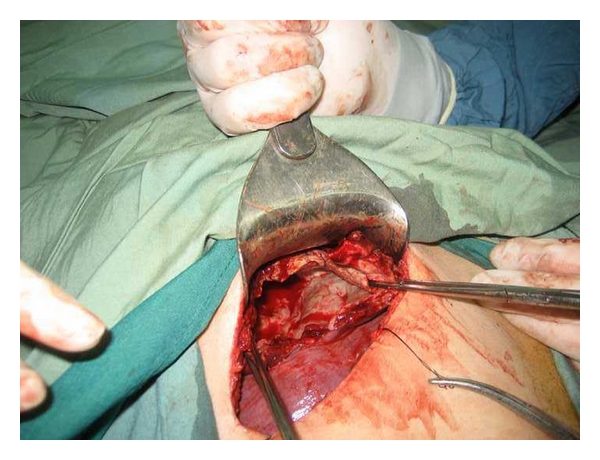
Residual cavity of a large hydatid cyst.

**Figure 2:**
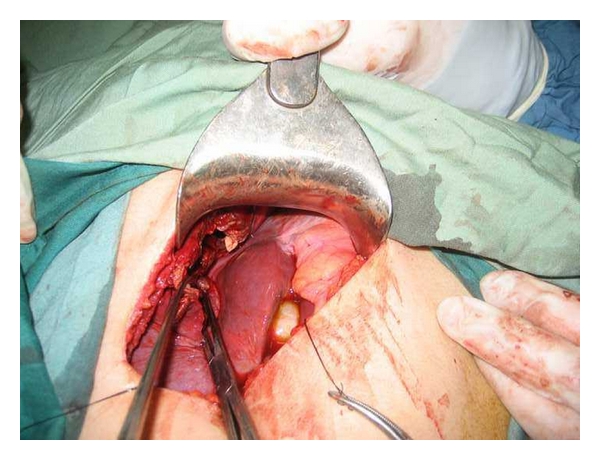
First layer closure of vest-over-pant method (medial cavity edge was approximated to cavity base).

**Figure 3:**
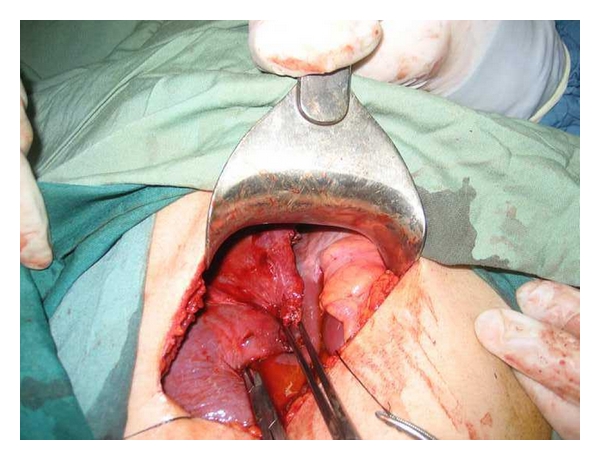
Second layer closure of vest-over-pant method (lateral cavity edge was approximated to dorsum of first layer).

In approach to liver hydatid cyst, we perform laparotomy with transverse right subcostal incision. Liver mobilization of right hepatic lobe exposing the bare area is performed by dissection of hepatic diaphragmatic attachments. In addition, division of the falciform ligament and peritoneal attachments to the left lateral segment are completed. Operative field is isolated with abdominal packs soaked with 0.5% AgNO3. After that, we proceed to a puncture aspiration of the cyst. Sterilization of cyst content is achieved by infusion of AgNO3 solution for 10 minutes. Each liver cyst is aspirated with a needle and cavity is filled with 0.5% AgNO3 for 10 minutes. Cyst fluid is aspirated again and then opened through a vertical incision to evacuate proligerous membrane. The cyst content is evacuated and cavity evaluated for biliary communications. For intramural cyst, we use cystectomy. Biliary communications are closed with direct suture. Then, the edges of one side of cyst are sutured to the base of the cavity using three to four mattress sutures with chromic 0 designed for liver suturing (Figure 1), and edges of other side of liver are overlapped on the dorsal part of previous layer using four to five mattress sutures (Figures 2 and 3). Therefore residual cavity dead space is obliterated with two surfaces of cavity. Drains are placed and abdominal wall is closed in layers.

## 3. Results

Fifty males were treated by our method. Ages ranged from 20 to 45 years. The clinical findings were abdominal mass in 20 (40%) cases and abdominal pain in 30 cases (60%). Cyst size was 82.2 ± 10.3 mm (55–145 mm). The described technique has been successfully used in 50 cases. All of the patients had single cyst. In 46 patients, cysts were located in right lobe. The average cyst volume was 423 ± 110 mL (300–650 mL). In two patients, biliary communications were closed by direct sutures. There was no intraabdominal sepsis, bile leakage, or hepatic necrosis. In follow-up ultrasound study, residual cavities were disappeared one month after operation. In one year followup, there was no fluid collection or recurrence of disease in ultrasound study.

## 4. Discussion

Hydatid cysts represent the liver stage of the dog tapeworm Echinococcus Granulosus. The disease is endemic in many countries such as Iran. The appropriate treatment of hydatid cyst is determined by several factors and is surgical or percutaneous drainage with intracystically injected scolicidal agents and chemotherapy. However there is considerable controversy about the management of the residual cavity. Many promising surgical techniques have been suggested through the years, but none has proven to be accurate enough, and their clinical relevance has been questioned. Cystectomy is considered the least traumatic method for hydatid cysts excision, but there is typically a larger residual cavity that can lead to a number of life-threatening complications such as suppuration, recurrence, and biliary fistula. Suture obliteration, omentoplasty, introflexion, double breasting, simple closure, deroofing, and tube drainage [5–11] are the most commonly used techniques for residual cavity management. Losanoff et al. [12] discussed various methods of residual cavity management techniques and their limitation. These limitations are inapplicability for extremely large cysts for omentoplasty, injury to blood vessels, and biliary tract in capitonnage, intestinal entrapment in the residual cavity in deroofing, and risk of spillage and anaphylactic shock in percutaneous aspiration under radiologic guidance. Losanoff used a variant of capitonnage method by manual compression of the healthy liver parenchyma and approximation of cavity edges with mattress sutures. For filling of dead space cavity, he used omentoplasty or gelatin sponge. In our study by using vest-over-pant method we obliterated the dead space without using of foreign materials or omentum. In this method there was no need for massive mobilization of liver and therefore, there was no additional damage to organ. It seems that this technique is a simple and effective method for residual cavity management in large hydatid cyst.
